# Advancing Atrial Fibrillation Research: The Role of Animal Models, Emerging Technologies and Translational Challenges

**DOI:** 10.3390/biomedicines13020307

**Published:** 2025-01-27

**Authors:** Monica Ferreira, Vera Geraldes, Ana Clara Felix, Mario Oliveira, Sergio Laranjo, Isabel Rocha

**Affiliations:** 1 Faculdade de Medicina, Universidade de Lisboa, 1649-004 Lisbon, Portugal; ferreira_t_monica@hotmail.com (M.F.); vgeraldes@medicina.ulisboa.pt (V.G.); mmartinsoliveira@gmail.com (M.O.); 2 Centro Cardiovascular da Universidade de Lisboa-CCUL, Universidade de Lisboa, 1649-004 Lisbon, Portugal; 3 Pediatric Cardiology Department, Hospital de Santa Marta, Unidade Local de Saúde de S. José, 1150-199 Lisbon, Portugal; ana.felix@ulssjose.min-saude.pt (A.C.F.); sergiolaranjo@gmail.com (S.L.); 4 Cardiology Department, Hospital de Santa Marta, Unidade Local de Saúde de S. José, 1150-199 Lisbon, Portugal; 5 CHRC, NOVA Medical School, Faculdade de Ciências Médicas, NMS, FCM, Universidade NOVA de Lisboa, 1169-056 Lisboa, Portugal

**Keywords:** atrial fibrillation (AF), animal models, pathophysiology, translational research, remodelling mechanisms

## Abstract

Atrial fibrillation (AF) is the most prevalent sustained cardiac arrhythmia, presenting a significant global healthcare challenge due to its rising incidence, association with increased morbidity and mortality, and economic burden. This arrhythmia is driven by a complex interplay of electrical, structural, and autonomic remodelling, compounded by genetic predisposition, systemic inflammation, and oxidative stress. Despite advances in understanding its pathophysiology, AF management remains suboptimal, with ongoing debates surrounding rhythm control, rate control, and anticoagulation strategies. Animal models have been instrumental in elucidating AF mechanisms, facilitating preclinical research, and advancing therapeutic development. This review critically evaluates the role of animal models in studying AF, emphasizing their utility in exploring electrical, structural, and autonomic remodelling. It highlights the strengths and limitations of various models, from rodents to large animals, in replicating human AF pathophysiology and advancing translational research. Emerging approaches, including optogenetics, advanced imaging, computational modelling, and tissue engineering, are reshaping AF research, bridging the gap between preclinical and clinical applications. We also briefly discuss ethical considerations, the translational challenges of animal studies and future directions, including integrative multi-species approaches, omics technologies and personalized computational models. By addressing these challenges and addressing emerging methodologies, this review underscores the importance of refining experimental models and integrating innovative technologies to improve AF management and outcomes.

## 1. Introduction

Atrial fibrillation (AF) encompassing its various forms, including paroxysmal, persistent, and long-standing persistent types, is the most prevalent sustained cardiac arrhythmia observed in clinical practice and its global incidence is increasing. This condition poses a substantial burden on the healthcare system because of its strong association with heightened morbidity, mortality and reduced quality of life. Characterised by disorganised, rapid electrical activation of the atria AF results in ineffective atrial contractions and an irregular ventricular response, thereby increasing the risk of ischaemic stroke, heart failure and systemic embolism [1,2,3].

The growing prevalence of AF can be attributed to demographic shifts towards older populations, improvements in diagnostic modalities, and the rising incidence of contributory factors such as obesity and hypertension [4,5]. Patients with AF are estimated to have a fivefold increased risk of ischaemic stroke and a twofold increase in both heart failure and all-cause mortality. Furthermore, AF considerably diminishes patient quality of life and escalates healthcare expenditure, with hospitalisations for AF-related complications constituting a major component of the associated economic burden [2,3].

Recognising the urgent need to translate experimental insights into clinical practice, the ESC Working Group on Cardiac Cellular Electrophysiology emphasises a nuanced understanding of the relative advantages and constraints of different experimental models. Although animal models are invaluable for dissecting the complex mechanisms underlying AF and for guiding therapeutic innovation, significant challenges remain in ensuring that findings derived from such models mirror human pathophysiology [6].

The pathophysiology of AF is multifaceted and involves intricate interactions among electrical, structural, and autonomic factors. Electrical remodelling, including ion-channel dysfunction, fosters a substrate predisposed to arrhythmogenesis. Structural alterations, such as atrial fibrosis and dilation, disrupt normal conduction pathways and disturbances in autonomic regulation, including both heightened sympathetic drive and altered parasympathetic tone, further contribute to AF initiation and maintenance [7,8]. Systemic inflammation, oxidative stress and genetic predisposition are increasingly recognised as pivotal contributors [9,10].

Recent advancements in genetic and molecular research have illuminated the hereditary underpinnings of AF and identified numerous genes and loci implicated in its development and progression. These discoveries have revealed distinct AF subtypes driven by a range of pathophysiological mechanisms, thereby informing the pursuit of personalised therapeutic strategies [10,11]. Concurrently, an enhanced understanding of the roles of inflammation and oxidative stress has paved the way for exploring novel anti-inflammatory and antioxidant treatments [9].

Nevertheless, critical questions remain regarding optimal management strategies. Balancing rate and rhythm control, refining anticoagulation approaches, and improving the safety and efficacy of therapeutic interventions are all pressing clinical needs [12]. Addressing these challenges requires robust translational pipelines that can transform mechanistic insights from experimental models into tangible clinical innovations.

Animal models have long served as an indispensable resource in this endeavour, facilitating the controlled reproduction of key arrhythmic substrates and enabling rigorous evaluation of disease progression and intervention efficacy. This review critically examines the spectrum of animal models employed in AF research and highlights their capacity to illuminate electrical, structural, and autonomic abnormalities. It also evaluates their inherent limitations and proposes strategies for enhancing their translational relevance, thereby narrowing the gap between preclinical discovery and clinical application.

## 2. Methods

This narrative review was conducted based on principles adapted from the PRISMA guidelines, aiming to ensure transparency and rigour in the selection and synthesis of the literature. The primary literature search was performed exclusively in the PubMed database, chosen for its comprehensiveness and relevance in the biomedical field. Additionally, Google Scholar was used as a complementary tool to identify further relevant articles, particularly those not indexed in PubMed. A total of 90 references were included, selected based on their relevance to the review’s theme and scientific quality, assessed by the originality of the data presented, their impact on AF understanding and methodological clarity. The inclusion criteria prioritised studies investigating electrophysiological, structural including cellular and autonomic mechanisms related to AF, regardless of the year of publication. Articles considered irrelevant to the central theme, duplicates or those with significant limitations in accessibility or methodological quality were excluded. Data extraction was performed qualitatively, highlighting the specific contributions of each animal model to understanding AF pathophysiology. The narrative synthesis emphasised the strengths and limitations of the models, as well as their implications for translational and therapeutic research.

## 3. Mechanistic Insights into Atrial Fibrillation

**Figure 1:**
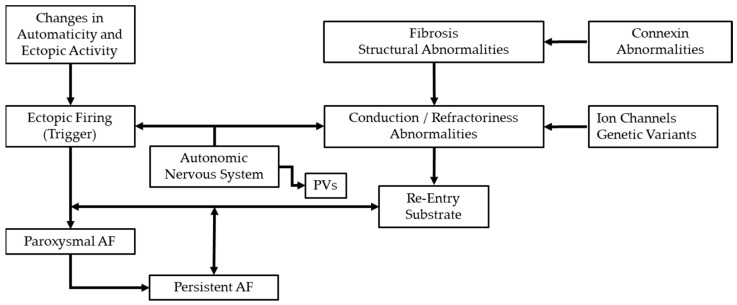
Mechanisms underlying AF Triggers and Persistence. This figure highlights the key mechanisms involved in atrial fibrillation.

Atrial fibrillation (AF) arises from a complex interplay between focal triggers such as ectopic foci and profound changes in the atrial substrate. These changes encompass electrical, structural, and autonomic remodelling, each contributing to both the initiation and persistence of AF. Figure 1 provides an overview of the interrelationships among these three facets of AF pathophysiology, illustrating how they collectively form a self-reinforcing cycle that sustains arrhythmia.

Experimental animal models have been instrumental in elucidating these interconnected mechanisms, as they allow controlled investigation of specific pathological features. While individual models often focus on distinct aspects such as electrical, structural, or autonomic remodelling, integrating findings across multiple models has been key to replicating the full complexity of human AF pathophysiology [6].

### 3.1. Electrical Remodelling

Electrical remodelling involves changes in the electrophysiological properties of the atrial myocardium, which increases the propensity for AF and favours its maintenance. These alterations occur at molecular, cellular, and tissue levels. A central hallmark is ion channel dysfunction, particularly the downregulation of L-type calcium currents (ICaLs), which shortens the atrial action potential duration and refractory period. This facilitates re-entry circuits and reduces the likelihood of spontaneous arrhythmia termination [13,14]. Studies employing rapid atrial pacing and genetically modified animal models have confirmed the role of such ion-channel alterations in promoting AF [6].

Simultaneously, increased potassium currents (e.g., IK1 and IKur) accelerates repolarization, thereby enhancing the substrate for re-entrant activity. Dysregulated calcium handling, including aberrations in ryanodine receptor function, contributes to additional triggers by promoting ectopic activity [15,16]. Conduction abnormalities also play a critical role: altered connexin expression and heterogeneous conduction velocities disrupt the uniform propagation of action potentials, creating a fertile environment for re-entry [7,17].

### 3.2. Structural Remodelling

Structural remodelling refers to anatomical and histopathological alterations in the atria that encourage AF persistence. Chronic haemodynamic stress resulting from conditions, such as hypertension, valvular disease, or heart failure, fosters fibrotic changes and atrial dilation. Animal models, particularly large mammalian species, have proven invaluable in recapitulating such advanced structural abnormalities, thereby offering insights into how they promote arrhythmogenesis [6].

Atrial fibrosis, characterised by excessive extracellular matrix deposition, impedes the orderly propagation of electrical impulses and enhances susceptibility to conduction block and re-entry. The activation of pro-fibrotic pathways, notably via transforming growth factor-β1 (TGF-β1) signalling, links chronic stress to the development of a pro-arrhythmic fibrotic substrate [18,19]. In addition, atrial dilation and myocyte hypertrophy, both of which are frequently induced by haemodynamic overload, alter chamber geometry and conduction pathways, further increasing vulnerability to AF [7,20,21].

### 3.3. Autonomic Remodelling

The autonomic nervous system exerts a profound influence on AF initiation and maintenance through modulation of atrial electrophysiology. Autonomic remodelling encompasses shifts in both parasympathetic and sympathetic balance. Animal models, particularly canine and porcine, have provided critical insights into these processes, including the role of intrinsic cardiac ganglia (ganglionated plexi) in integrating autonomic inputs [6,22,23].

Parasympathetic stimulation, primarily via the vagus nerve and acetylcholine release, shortens the atrial effective refractory period by enhancing I_K(ACh), thus creating regions of heterogeneous refractoriness conducive to re-entry [24,25]. Conversely, sympathetic hyperactivity increases beta-adrenergic tone and augments calcium influx, fostering triggered activity and ectopic firing [16]. Chronic sympathetic stimulation further amplifies oxidative stress and calcium overload, promoting both structural and electrical remodelling [20,21]. Through these combined effects, autonomic influences reinforce arrhythmogenic substrates and facilitate AF persistence.

### 3.4. Interplay Between Mechanisms

Electrical, structural, and autonomic remodelling are not isolated phenomena; rather, they form a dynamic and interdependent triad. For instance, fibrotic remodelling can create substrates that magnify the impact of autonomic triggers, whereas electrical disturbances can enhance the response to both structural and autonomic perturbations. Collectively, these mechanisms constitute a self-perpetuating arrhythmogenic cycle. Post-ablation studies examining alterations in autonomic function highlight the integral role of neuromodulation in disrupting this cycle [6,15,21,26,27,28].

A comprehensive understanding of these interactions is vital for developing effective therapeutic strategies. By exploring these processes in animal models, researchers can dissect the complexity of AF pathophysiology and identify targets to break the vicious cycle of sustaining this challenging arrhythmia.

## 4. Animal Models in Mechanistic Studies

Animal models are indispensable for elucidating the individual and combined contributions of electrical, structural, and autonomic mechanisms to AF [29,30,31]. Rodents are frequently employed to investigate the molecular foundations of electrical remodelling, including ion channel dysfunction and abnormalities in calcium handling, owing to their genetic tractability and cost-effectiveness. In contrast, larger animals, such as goats and pigs, whose cardiac anatomy and physiology more closely resemble those of humans, are better suited for exploring the structural and autonomic dimensions of AF. For instance, sustained rapid atrial pacing in goats reproducibly induces atrial fibrosis and dilation, thereby mirroring advanced stages of the human condition [7].

Despite their value, each animal model has inherent advantages and limitations, particularly when attempting to recapitulate the complexity of human AF. Species-specific differences in anatomy, physiology, and electrophysiology influence baseline heart rates, action potential characteristics, and reproducibility and maintenance of AF. These variations must be carefully considered when interpreting the results and translating the findings into clinical practice. Table 1 provides a comparative overview of the commonly employed animal models, detailing AF induction methods, experimental techniques, and primary outcome measures in mechanistic studies. Such comparisons underscore translational hurdles and highlight the importance of selecting the most appropriate model for investigating specific aspects of AF pathogenesis [6,32]. In addition, emerging technologies, including optogenetics and high-resolution cardiac mapping, have significantly advanced AF research by enabling precise spatial and temporal characterisation of both electrical and autonomic activity. Such innovations have yielded unprecedented insights into AF initiation and maintenance, thereby enhancing our understanding of the multifactorial nature of arrhythmia and guiding the development of more targeted interventions [33].

### 4.1. Rodents (Mice and Rats)

Rodents, particularly mice and rats, have long been integral to AF research owing to their genetic tractability, cost-effectiveness and practicality in laboratory settings. These models are invaluable for dissecting the molecular and cellular mechanisms underlying AF, providing insights that are often difficult to achieve in larger animals or in human studies. Mouse models typically rely on significant genetic or physiological alterations to induce AF, which often leads to pronounced cardiac dysfunction and atrial remodelling [32,34,35]. As spontaneous AF is rare in mice, programmed electrical stimulation is commonly used to induce short-lived AF episodes. Although these approaches successfully replicate key electrical and structural changes, they generally fail to mimic human-specific triggers such as pulmonary vein ectopy. Despite their many advantages, the translational relevance of rodent models remains limited.

Rodent studies have offered foundational insights into the arrhythmogenic substrates central to AF pathophysiology, particularly in relation to electrical remodelling, ion channel dysfunction and calcium-handling abnormalities [6,36]. For example, investigations in rodents have demonstrated the pivotal role of altered L-type calcium currents (ICaL) and augmented potassium currents (IK1, IKur) in promoting electrical instability [7,9].

One of the greatest strengths of rodent models is their unparalleled suitability for genetic manipulation. The availability of transgenic, knockout and knock-in technologies allows researchers to isolate and investigate the specific molecular pathways implicated in AF pathogenesis. Mutations in key ion channels, including Nav1.5 (SCN5A) and Kir2.1 (KCNJ2), previously linked to familial AF, have been successfully studied in these organisms. Transgenic rodents overexpressing atrial-selective ion channels or calcium-handling proteins have provided crucial insights into the origins of electrical remodelling, while knockout models have clarified the roles of particular genes in atrial excitability and arrhythmia susceptibility [11,14,37].

A notable example is the cardiac-specific liver kinase B1 (LKB1) knockout mouse model, which progresses from sinus rhythm to persistent AF, closely mirroring the natural history of human disease [34]. This model highlights how evolving inflammatory atrial cardiomyopathy, accompanied by electrical and structural remodelling (including bi-atrial enlargement, fibrosis and cardiomyocyte loss), drives AF development. The interplay between inflammation, reactive oxygen species and disrupted connexin-mediated gap junction coupling (notably connexins 40 and 43) underscores the multifactorial nature of AF pathogenesis [38]. These mechanistic insights have guided the identification of potential therapeutic targets, reinforcing the translational promise of rodent models [11,14,32,39,40,41].

Recent metabolomic studies have explored non-traditional AF promoters using rodent models, including the influence of sleep deprivation, obesity and the gut microbiome [42,43,44,45]. For instance, high-fat diets or microbiome transplantation from patients with AF significantly increased AF episodes in mice, whereas sleep deprivation did not. Obesity-related studies have revealed impaired glycolysis, elevated β-oxidation, lipid accumulation and raised branched-chain amino acid levels. However, physiological differences such as variations in epicardial fat distribution limit the direct application of these findings to humans. The inconsistent reporting of sample sizes and metabolites across studies also constrains the reliability and translational utility of such approaches.

Rats share many of the same advantages and limitations as mice, although they have historically been employed less extensively in AF research. However, certain rat models, such as the spontaneously hypertensive rat, have proven valuable for examining atrial remodelling and AF predisposition. Acquired AF triggers, including intensive exercise, sleep apnoea, myocardial infarction and pulmonary hypertension, have also been investigated in rats [41,46]. Nevertheless, their overall contribution to AF research has been relatively limited compared to mouse models [47,48,49].

Rodents are highly cost-effective for large-scale investigations, with low maintenance costs, short reproductive cycles and are suitable for longitudinal studies. These attributes facilitate rapid testing of new antiarrhythmic compounds targeting atrial-selective ion channels, bolstering their role in preclinical drug discovery [9].

However, the interpretability of rodent data is hindered by stark physiological differences compared with humans, including higher heart rates, differing ion channel profiles and minimal baseline atrial fibrosis. Tools routinely used in clinical practice, such as catheters and pacemakers, are often unsuitable for such small animals, necessitating customised devices and limiting the scope of invasive electrophysiological assessments. Although optical mapping techniques have improved arrhythmia characterisation ex vivo, the small size of rodent atria limits the extent to which fibrillatory conduction and re-entrant dynamics can be faithfully replicated [8]. Interventions like angiotensin II infusion or rapid atrial pacing are sometimes required to induce human-like structural changes, such as fibrosis and chamber dilation [10]. As a result, findings from rodents must be interpreted cautiously and complementary studies in larger animals are often necessary to enhance translational relevance [6].

Technological advancements have expanded the potential of rodent models. Optogenetics enables precise spatiotemporal control of ion channels, facilitating detailed mapping of arrhythmogenic circuits, whereas CRISPR-Cas9 genome editing accelerates the creation of highly specific models tailored to test specific hypotheses. These innovations promise to improve the fidelity of rodent models and offer richer insights into the complex aetiology and maintenance of AF [33].

### 4.2. Larger Animal Models (Rabbits, Dogs, Goats, Pigs, Sheep, Horses, and Non-Human Primates)

Larger animal models, such as rabbits, dogs, goats, pigs, sheep, horses and non-human primates, are integral to AF research as they bridge the gap between rodent studies and clinical applications in humans. Their anatomical and physiological features closely approximate those of the human heart, facilitating the investigation of structural and autonomic remodelling, which are key dimensions that are less accessible in smaller models. These systems enable the study of chronic AF mechanisms, guide therapeutic development and advance translational research [50]. Table 2 summarises the principal characteristics of these models, including their advantages, limitations and specific research applications.

#### 4.2.1. Rabbits

Rabbits occupy a niche as intermediate models, combining the relatively straightforward handling of smaller species with greater physiological relevance than that of rodents. Their atrial size and heart rate were closer to human values, thus improving data extrapolation. In particular, rabbit atrial myocytes exhibit calcium-handling properties closely aligned with those of humans, making rabbits invaluable for studying calcium dynamics and the early phases of electrical remodelling [8]. They have proven useful in exploring cardiac repolarization and diverse acute AF induction methods, including acetylcholine stimulation, rapid pacing and programmed electrical stimulation [51]. Studies in rabbits have also shed light on how autonomic modulation involving both sympathetic and parasympathetic inputs influences AF inducibility [25,52]. Despite these strengths, rabbits have relatively limited genetic tools, shorter lifespans and species-specific autonomic responses that differ from those in humans. Consequently, while they are valuable for probing conduction heterogeneities and sarcoplasmic reticulum calcium release [9], these constraints must be considered when interpreting findings [7].

#### 4.2.2. Dogs

Among larger models, dogs provide an unparalleled degree of anatomical and physiological similarity to humans, particularly in terms of atrial structure and autonomic innervation [6]. They can sustain AF induction through rapid atrial pacing, enabling investigations into disease progression, autonomic remodelling and therapeutic interventions [53,54,55]. Canine models are especially useful for examining vagal stimulation, ganglionated plexi activation and their contribution to AF initiation and maintenance. They also help elucidate the links between AF and central nervous system disorders [22,56,57]. However, ethical considerations, high maintenance costs and response variability pose major challenges. Nonetheless, dogs remain a pivotal model for testing catheter ablation techniques and pharmacological strategies targeting autonomic pathways [58].

#### 4.2.3. Goats

Goats, featuring larger atria and capacity for sustained AF induction, represent a robust model for studying long-term structural remodelling. Chronic pacing protocols induce atrial dilation and fibrosis, mimicking the advanced stages of human AF [18,32,59]. Goats tolerate AF relatively well, facilitating prolonged studies of disease progression and evaluation of surgical or catheter-based therapies targeting fibrotic substrates [18]. Although the limited availability of genetic tools and high costs limit the widespread adoption of goat models, their reproducibility and close approximation of persistent human AF make them invaluable in mechanistic research.

#### 4.2.4. Sheep

Sheep are frequently employed in AF research because of their capacity to model various aspects of human AF pathophysiology. Although AF rarely occurs spontaneously in sheep, experimental protocols such as atrial tachypacing reliably induce electrical and structural remodelling. These models have revealed the interplay among fibrosis, atrial adipose tissue, inflammation and arrhythmogenesis [60]. Investigations have demonstrated that interventions like galectin-3 inhibition or eplerenone treatment can reduce AF burden [61]. Age-dependent changes in calcium handling and atrial alternans are also evident in sheep, while the unique coronary anatomy permits exploration of the impact of atrial ischaemia on AF. Obesity and hypertension studies in sheep highlighted fibrosis, epicardial fat infiltration and slowed conduction as AF risk factors [62]. Furthermore, sheep pulmonary veins structurally differ from but functionally resemble human PVs, clarifying their role in AF initiation. However, the high cost of housing and limited physiological similarity compared to pigs constrain the broader application of sheep models.

#### 4.2.5. Pigs

Pigs may offer the highest translational relevance because of their cardiac anatomy, physiology and electrophysiology, which closely parallels those of humans. Their large atrial size permits the use of human-scale catheters and advanced imaging tools, such as three-dimensional electroanatomic mapping [63]. Pigs are a preferred model for testing catheter-based ablation techniques and understanding the interplay between myocardial ischaemia and atrial remodelling [64,65,66]. Sustained AF in pigs induces changes in refractory periods, calcium homeostasis, and the expression of key proteins such as CaMKII, RyR2, and Cx43. Gene therapy targeting Cx43 can restore its expression and prevent persistent AF. Histologically, pigs develop atrial and ventricular hypertrophy, fibrosis and apoptosis, reflecting the progression to severe congestive heart failure [32,67,68]. Nevertheless, the high costs, limited coronary collateralization [51] and associated risks of ventricular fibrillation during coronary occlusion constrain the widespread use of pigs. Despite these challenges, pigs remain the cornerstone of translational AF research.

#### 4.2.6. Horses

Horses present a unique opportunity to study atrial conduction pathways at a scale approximating human atrial size as well as their multiple and larger pulmonary veins [69,70]. This structural similarity enables the detailed mapping of conduction and investigation of fibrotic remodelling [71]. However, ethical challenges, substantial costs and limited accessibility restrict the widespread use of equine models [72].

#### 4.2.7. Non-Human Primates

Non-human primates offer the closest physiological and anatomical resemblance to humans, making them highly valuable for chronic AF studies and the testing of advanced therapies. Despite their translational potential, the use of primates is heavily limited by ethical concerns, high costs and limited availability [72].

## 5. Comparative Analysis of Models

Selecting an appropriate animal model is central to advancing our understanding of AF pathophysiology as different species capture distinct aspects of this complex arrhythmia. Each model offers unique strengths and limitations that influence its applicability to studies of electrical, structural, and autonomic remodelling. In general, a gradient of translational relevance emerges across species, shaped by considerations of cost, physiological similarity to humans and feasibility of experimental interventions (Table 3).

Rodents (mice and rats) are invaluable for probing the molecular and cellular underpinnings of AF, particularly in relation to electrical remodelling. They provide critical insights into ion channel dysfunction and abnormal calcium handling [8]. However, their capacity to replicate the structural changes inherent to human AF, such as extensive atrial fibrosis and dilation, is limited. Similarly, rodents’ high baseline heart rates and comparatively simple autonomic innervation reduce their utility in autonomic remodelling studies [7]. Despite these shortcomings, the low cost, ease of genetic manipulation, and rapid experimental throughput render rodents a valuable platform for early-stage research [9,40].

Rabbits occupy the middle ground between rodents and larger animals, offering improved physiological relevance and human-like electrophysiological properties. Their atrial size and heart rate are closer to those of humans, enabling more meaningful investigations into electrical remodelling and conduction heterogeneities [8,73]. Although rabbits can model some aspects of structural and autonomic remodelling, their limited genetic tools and moderate translational potential limit their broader application. Nevertheless, moderate maintenance costs and improved physiological relevance over rodent’s place rabbits as a practical intermediate model [7].

Historically, dogs have been central to AF research because of their close anatomical and physiological resemblances to the human heart. Sustained AF induction via rapid atrial pacing permits comprehensive exploration of electrical remodelling, while intrinsic cardiac ganglia and pulmonary vein innervation closely mirror human autonomic networks [22,58]. This makes canine models well-suited for investigating both autonomic remodelling and structural changes, including fibrosis and dilation. Its high translational relevance renders dogs invaluable for testing therapeutic interventions. However, ethical concerns, elevated costs and inter-individual variability remain significant barriers to routine use [40,57,74].

Goats excel in modelling structural remodelling. Their large atria, capacity for sustained AF induction and tolerance to long-term pacing facilitate detailed examination of fibrosis and atrial dilation [59]. Although goats can also model electrical and autonomic remodelling reasonably well, their utility is limited by a lack of genetic tools and the high maintenance cost [18]. Goats remain the model of choice for investigating chronic structural changes and assessing surgical or catheter-based approaches that target fibrotic AF substrates.

Pigs are widely considered to have translational relevance in AF research. Their cardiac anatomy, physiology and sophisticated autonomic innervation closely parallel human conditions, making them ideal for studying all facets of AF pathophysiology—electrical, structural and autonomic remodelling [70]. Pigs are uniquely suited for evaluating advanced imaging techniques, catheter ablation strategies and novel therapeutic interventions [23,64,75]. Nevertheless, the high cost, ethical considerations and logistical complexities associated with pig models limit their widespread use [40].

In summary, no single animal model perfectly recreates all aspects of human AF and the choice of model should reflect the specific research objectives. Early stage molecular and cellular studies often favour rodents, whereas investigations into the structural and autonomic complexities of AF may require larger models such as goats or pigs. By carefully weighing the strengths and limitations of each species, researchers can strategically select the most suitable model to advance AF research from the bench to bedside.

## 6. Emerging Approaches

Recent advances in AF research are reshaping our understanding of the condition and expanding the repertoire of tools available for basic, translational and clinical investigations. By integrating cutting-edge methodologies ranging from precision optical interventions to complex computational analyses, these emerging strategies promise to refine our understanding of AF mechanisms and accelerate the development of more effective, personalised therapies.

A key development is optogenetics, which employs genetically encoded light-sensitive ion channels to modulate atrial electrophysiology with extraordinary spatial and temporal precision. Researchers can now illuminate specific atrial regions to dissect re-entrant pathways and examine how autonomic innervation contributes to AF initiation and perpetuation, facilitating previously unattainable insights into arrhythmogenic substrate [33,76,77].

Building on these refined experimental manipulations, advances in three-dimensional cardiac mapping techniques and imaging modalities have increased the study of AF to new levels of anatomical and functional details. Electroanatomic mapping systems, when integrated with MRI and other imaging modalities, enable the precise identification of arrhythmic foci, conduction heterogeneities and fibrotic substrates. These technologies do not merely delineate abnormalities; coupled with computational modelling, they form predictive frameworks that can guide patient-specific interventions and enhance the efficacy of therapeutic approaches [40,63,78,79,80].

The complexity of the AF requires an equally complex approach. Integrative models, which incorporate electrical, structural and autonomic elements, bridge the gaps left by reductionist platforms. By simulating the multifactorial environment of the human atria, encompassing fibrosis induction, autonomic stimulation and altered ion channel function, these models more faithfully recreate the spectrum of AF pathophysiology. Such holistic frameworks also facilitate the testing of multimodal interventions, such as combined pharmacological and catheter-based therapies, and can be further enhanced by computational modelling to inform clinical decision-making [6,20,31,80,81].

While integrative experimental models replicate complexity, the rise of artificial intelligence and machine learning has brought data-driven precision to AF research. These algorithms can synthesise genetic, imaging and electrophysiological datasets to identify previously unrecognised biomarkers, predict AF recurrence and tailor interventions to individual patient profiles [4,40,82,83]. Such computational intelligence complements experimental insights and pushes the field towards personalised medicine as shown in the computationally guided, personalised targeted ablation approach outlined by Boyle et al. [84]. A recent study by Sakata et al. [85] utilized a personalised digital-twin approach to optimize the distribution of ablation lesions, which highlights the potential of computational models in refining AF treatments and preventing complications such as post-ablation atrial tachycardia.

The recognition that AF is influenced not only by electrical irregularities, but also by mechanical factors, has sparked interest in electromechanical coupling studies. Ultrasound-based strain imaging and pressure-volume loop analyses now provide insight into how mechanical deformation interacts with altered conduction to sustain AF. These findings underscore the importance of addressing both electrical and mechanical dysfunction, advancing beyond single-dimension interventions [31].

At the molecular and cellular levels, single-cell and spatial transcriptomics have revolutionised our perspective on atrial heterogeneity. These advanced genomic technologies allow high-resolution mapping of gene expression profiles, exposing cell type-specific contributions to AF, including those within fibrotic, inflammatory and electrophysiologically distinct regions. Such insights may lead to highly targeted therapies aimed at distinct cellular subpopulations [9,82,83,86].

Simultaneously, organoids and tissue engineering are forging new links between fundamental biology and clinical relevance. Human pluripotent stem cell-derived atrial organoids and engineered atrial tissues recapitulate key aspects of human atrial physiology, creating controlled environments in which to study AF pathology and test for potential treatments. These bioengineered systems can also be patient-specific, enabling drug screening and mechanistic studies that honour individual variability [76,77,87,88,89]. A study by Azzolin et al. [90] compares personalised ablation strategies to conventional approaches, highlighting the potential of tailored therapies to improve outcomes in atrial fibrillation treatment.

Finally, all of these methodologies converge in the realm of in silico models, where computational simulations integrate imaging, electrophysiological and genetic data. This approach reduces reliance on animal models and invasive procedures, offering an ethically advantageous and cost-effective platform for hypothesis testing and therapeutic prediction. In silico models can test how different pharmacological, electrical, or structural interventions might influence AF outcomes, streamlining the pathway to clinical application [33,40,82,83,91,92,93]. Together, these emerging approaches shape a more integrated and nuanced understanding of AF. By capitalising on precise experimental control, advanced imaging, intelligent data analysis, and increasingly faithful disease models, AF research is moving closer to robust patient-centred therapies. In doing so, it lays the foundation for transforming AF management from a predominantly reactive endeavour into a preventative, personalised and mechanistically informed discipline.

## 7. Challenges and Future Directions

Despite considerable advances in AF research and the development of diverse experimental models, significant challenges remain. Enhancing the translational impact of current approaches, ensuring ethical and economic feasibility, and refining the alignment of models with human pathophysiology are key areas that require urgent attention. Meeting these challenges will depend on integrating emerging technologies and methodologies, ranging from advanced computational modelling to human-derived tissues, into a more unified research framework.

One of the most pressing issues is the validation of experimental models using human clinical data. While animal studies have yielded invaluable insights into AF pathophysiology, substantial physiological differences persist, particularly in relation to atrial size, ion channel expression and autonomic innervation [33,72,73,82,91,92,93]. Advanced computational models incorporating multiscale data from ion channels to organ-level dynamics are becoming indispensable tools for validating preclinical findings. By simulating patient-specific conditions, these in silico platforms can bridge the gap between experimental outcomes and clinical realities, and guide researchers in refining model systems that more accurately replicate human AF.

Ethical considerations also shape the future of AF modelling. Large animal models such as dogs, goats, and pigs provide invaluable access to the complex structural and autonomic features of arrhythmia, but their use is limited by both ethical and logistical constraints. The increasing reliance on human-derived systems, including organoids, engineered tissues, and organ-on-chip platforms, offers a more ethically sound and cost-effective alternative. These innovative approaches can recapitulate key aspects of human AF pathophysiology without the moral dilemmas associated with large-animal experimentation [40,82,83]. Incorporating these biological models into computational workflows will further enhance the predictive power and reduce the need for extensive animal use [6].

Bridging the translational gap between preclinical research and clinical applications remains an ongoing challenge. Prioritising endpoints that mirror human disease characteristics, such as atrial fibrosis, conduction abnormalities, and patient-specific electrophysiological profiles, can better inform therapeutic development [9,31,82,83]. Integrating computational simulations and advanced imaging can help to identify conserved molecular and cellular pathways across species. Such integrative strategies, as highlighted by Heijman et al. [80], hold the promise of accelerating the translation of findings by enabling virtual clinical trials that predict patient responses to new treatments.

Several interlinked strategies suggest themselves as pathways to overcome these challenges. Comparative analyses that examine AF mechanisms across multiple animal models and human data can reveal both convergent and divergent processes, thereby guiding the selection of the most suitable model for each research question. A broader integration of omics technologies, including genomics, transcriptomics, proteomics, and metabolomics, can identify novel biomarkers and therapeutic targets, paving the way for personalised medical approaches tailored to each patient’s molecular signature [4,40,82,83]. Moreover, the refinement of patient-specific in silico models based on imaging, genetic, and electrophysiological data will enhance the precision of predictions regarding AF progression and treatment outcomes, thereby reducing the need for extensive animal experimentation [33,82,83].

Ultimately, animal models, which are historically foundational and rich in investigative potential, must be viewed as part of a broader ecosystem of research tools. These models are invaluable for dissecting disease triggers, genetic factors and molecular mechanisms; however, they remain intrinsically limited by physiological differences from humans and the complexity of AF as a multifactorial disorder. No single system can reproduce the full spectrum of human AF. Instead, the forward path lies in harnessing the complementary strengths of multiple approaches. The integration of animal studies with human-derived tissue platforms, in silico simulations, advanced imaging, and omics-based profiling will create a more nuanced, ethically sound and clinically relevant research paradigm. As these innovative methodologies continue to mature, they will not only reduce reliance on animal models and their associated costs but will also bring us closer to the ultimate goal of effective, personalised, and mechanism-informed therapies that improve the lives of patients with AF worldwide.

## Figures and Tables

**Table 1:** Experimental Approaches Across Animal Models in AF Research. This table highlights the commonly used animal models in AF research, detailing the methods employed to induce AF, techniques applied to study the disease, and primary outcomes measured.

Model	Induced-AF Method	Techniques Used	Outcomes Measured
Rodents (Mice, Rats)	Rapid atrial pacing	Genetic manipulation, optogenetics	Ion channel dysfunction, calcium handling.
Rabbits	Atrial burst pacing	Calcium imaging, vagal stimulation	Conduction heterogeneities.
Goats	Long-term pacing	3D mapping, fibrosis markers	Fibrosis, dilation.
Pigs	Ischemia, catheter-based pacing	Advanced imaging, catheter ablation	Electrical and autonomic remodelling.

**Table 2:** Emerging Technologies in AF research. This table summarises the key emerging technologies that have advanced the study of AF. Each technology offers unique capabilities, addressing the limitations of traditional approaches, while enabling detailed exploration of AF mechanisms and potential therapies.

Technology	Description	Key Advantages	Limitations	Applications
Optogenetics	Precise control of ion channel activity using light-sensitive proteins.	High spatial/temporal precision.	Requires genetic modification; complex setup.	Mapping re-entrant circuits.
3D Cardiac Mapping	Visualization of conduction pathways and arrhythmic foci.	High-resolution imaging; predictive insights.	Costly and technically demanding.	Studying structural substrates.
Computational Models	In silico simulations of AF mechanisms.	Cost-effective; ethical; patient-specific.	Data quality-dependent; validation needed.	Drug testing, virtual trials.

**Table 3:** Comparative characteristics of animal models used in AF research. This table summarises the key differences among various animal models used in AF research, with each model contributing uniquely to the understanding of AF mechanisms, from molecular pathways to structural remodelling and therapeutic testing. HR: heart rate.

Animal Model	Resting HR (bpm)	Heart Weight	Advantages	Limitations	Applications in AF Research
Mice	600–800	~0.15 g	Genetically tractable (knockout/knock-in models); Fast reproduction; Cost-effective	Extremely fast heart rate; Small atrial size; Limited fibrosis and dilation	Ion channel dysfunction; Ca^2+^ handling abnormalities; Early-stage electrical remodelling
Rats	~400	~0.5 g	Larger size than mice; Suitable for pharmacological testing; Longer studies possible	Fast heart rate; Limited translational relevance due to small atrial dimensions	Calcium dynamics; Drug testing; Molecular mechanisms of AF
Rabbits	130–300	3–5 g	Moderate heart rate closer to humans; Atrial calcium dynamics resemble humans	Limited genetic tools; Short lifespan; Limited fibrosis development	Calcium handling studies; Early electrical remodelling
Guinea Pigs	~300	~1–2 g	Longer action potential duration; Intermediate heart rate	Limited genetic and molecular tools; Underutilized in AF research	Atrial action potential investigations; Electrophysiology studies
Dogs	80–160	100–120 g	Closely mimic human cardiac and autonomic systems; Reliable sustained AF	Ethical concerns; High maintenance costs; Experimental variability	Autonomic remodelling; Ganglionated plexi studies; Catheter ablation testing
Goats	84–96	~100–150 g	Capable of long-term pacing-induced AF; Atrial size similar to humans	Limited genetic tools; Expensive maintenance	Persistent AF models; Structural remodelling studies; Fibrosis induction
Pigs	68–100	~250–350 g	Human-like cardiac anatomy and physiology; Suitable for catheter-based studies	Ethical considerations; High costs; Limited transgenic models	Structural and autonomic remodelling; Catheter ablation and mapping;
Sheep	76–91	~200–250 g	Large atrial size for sustained AF; Chronic AF with fibrosis and dilation	Costly maintenance; Ethical concerns; Less availability	Chronic AF studies; Hemodynamic overload; Structural remodelling;
Horses	42–200	~3–5 kg	Large atrial size for modelling global conduction pathways; Closely resembles human atrial dynamics	Ethical challenges; Limited accessibility; High costs	Mapping atrial propagation pathways; Fibrosis and structural remodelling;
Non-human Primates	60–90	~250–400 g	Closest physiological and anatomical similarity to humans; Relevant for chronic AF studies	Significant ethical barriers; Very high costs; Rarely used in AF studies	Translational research; Advanced therapeutic testing; Chronic AF models
